# Human Neurons Form Axon-Mediated Functional Connections with Human Cardiomyocytes in Compartmentalized Microfluidic Chip

**DOI:** 10.3390/ijms23063148

**Published:** 2022-03-15

**Authors:** Martta Häkli, Satu Jäntti, Tiina Joki, Lassi Sukki, Kaisa Tornberg, Katriina Aalto-Setälä, Pasi Kallio, Mari Pekkanen-Mattila, Susanna Narkilahti

**Affiliations:** 1 Heart Group, Faculty of Medicine and Health Technology, Tampere University, 33520 Tampere, Finland; martta.hakli@tuni.fi (M.H.); katriina.aalto-setala@tuni.fi (K.A.-S.); mari.pekkanen-mattila@tuni.fi (M.P.-M.); 2 Neuro Group, Faculty of Medicine and Health Technology, Tampere University, 33520 Tampere, Finland; satu.jantti@tuni.fi (S.J.); tiina.joki@wikli.fi (T.J.); 3 Micro- and Nanosystems Research Group, Faculty of Medicine and Health Technology, Tampere University, 33720 Tampere, Finland; lassi.sukki@tuni.fi (L.S.); kaisa.tornberg@tuni.fi (K.T.); pasi.kallio@tuni.fi (P.K.); 4 Heart Hospital, Tampere University Hospital, 33520 Tampere, Finland

**Keywords:** neuron, cardiomyocyte, coculture, axon-mediated, functional interaction, human-induced pluripotent stem cell, organ-on-chip, microfluidic chip

## Abstract

The cardiac autonomic nervous system (cANS) regulates cardiac function by innervating cardiac tissue with axons, and cardiomyocytes (CMs) and neurons undergo comaturation during the heart innervation in embryogenesis. As cANS is essential for cardiac function, its dysfunctions might be fatal; therefore, cardiac innervation models for studying embryogenesis, cardiac diseases, and drug screening are needed. However, previously reported neuron-cardiomyocyte (CM) coculture chips lack studies of functional neuron–CM interactions with completely human-based cell models. Here, we present a novel completely human cell-based and electrophysiologically functional cardiac innervation on a chip in which a compartmentalized microfluidic device, a 3D3C chip, was used to coculture human induced pluripotent stem cell (hiPSC)-derived neurons and CMs. The 3D3C chip enabled the coculture of both cell types with their respective culture media in their own compartments while allowing the neuronal axons to traverse between the compartments via microtunnels connecting the compartments. Furthermore, the 3D3C chip allowed the use of diverse analysis methods, including immunocytochemistry, RT-qPCR and video microscopy. This system resembled the in vivo axon-mediated neuron–CM interaction. In this study, the evaluation of the CM beating response during chemical stimulation of neurons showed that hiPSC-neurons and hiPSC-CMs formed electrophysiologically functional axon-mediated interactions.

## 1. Introduction

Cardiac function is modulated by the sympathetic and parasympathetic branches of the cardiac autonomic nervous system (cANS), which can stimulate or inhibit, e.g., the heart rate and contraction force. In vivo, neuronal axons innervate cardiac tissue [1,2,3], while neuronal somas reside in either central or peripheral nervous system (CNS and PNS, respectively) [1]. During embryonic cardiac innervation, neurons and cardiomyocytes (CMs) undergo comaturation, where the growth and transmission properties of the innervating neurons are regulated by signals from cardiac tissue and CM maturation is influenced by neuronal signals [2]. As cANS plays a crucial role in cardiac function, the dysregulation of this system due to pathological conditions, such as arrhythmias or ischemic heart disease, can be fatal [4]. Furthermore, the modulation of cardiac function via drugs targeting the cANS can be advantageous regarding drug development for cardiovascular diseases [5]. For example, therapeutic targeting of neurons to improve cardiac outcome after heart failure is important; however, studying this requires human cell-based models as drug responses and disease mechanisms between animal and human cells can be different [3,6,7]. Moreover, detection of adverse effects in cardiac tissue or the cANS is important to ensure drug safety [3]. Thus, relevant but reductionist models for human cardiac innervation are needed, where organ on a chip models could reduce the gap between animal models and human diseases [8,9].

Previously reported coculture devices used for neurons and CMs were compartmentalized polydimethylsiloxane (PDMS)-based chips that allowed the isolation of neuronal somas from the CMs and restricted cell-to-cell interactions to axons extending through microtunnels between the compartments [10,11,12]. Chips were integrated with microelectrode arrays (MEAs), allowing electrical stimulation and functional evaluation of the neurons and CMs. Studies have shown the formation of functional connections between rat superior cervical ganglion neurons and rat ventricular CMs [10], rat superior cervical ganglia neurons as sympathetic neurons, rat intracardiac ganglia neurons as parasympathetic neurons and rat atrial CMs [11], and rat primary sympathetic neurons and human-induced pluripotent stem cell (hiPSC)-derived CMs [12]. Moreover, a coculture of hiPSC-derived PNS neurons with hiPSC-CMs in a compartmentalized chip has been reported; however, only evidence of physical neuromuscular connections was shown, without further evaluation of electrophysiological functionality [13]. hiPSC-neurons and hiPSC-CMs have also been cocultured without restrictions of cell-to-cell contacts, showing the formation of functional interactions between the cell types [14,15]. Previously reported neuron–CM coculture models have not shown functional connections between human neurons and CMs via axon-mediated interactions alone. However, human cell-based models are desperately needed, e.g., in drug development, where animal and animal cell models are frequently used with poor predictive power for human response [6]. Possible differences in animal and human disease mechanisms complicate the translation of results to humans [16].

Primary human neurons and CMs are difficult to harvest, while hiPSCs can be easily differentiated into neurons and CMs in large quantities [6]. As many diseases have multicellular contributions, monocultures may not recapitulate the disease mechanism faithfully [17]. This study aimed to develop an advanced, functional, and completely human-based cardiac innervation on a chip for studying cardiac innervation and neuron–CM interactions. The coculture of spontaneously electrically active hiPSC-neurons [18] and hiPSC-CMs [19,20] was performed in a previously developed 3D3C chip [21,22] containing three separate culture compartments connected via microtunnels allowing only axonal connections between the compartments. In this study, we show that the 3D3C chip enables long-term coculture of the neurons and CMs in their respective media, with the neurons forming axon-mediated interactions with the cardiomyocytes, mimicking the in vivo situation. This study shows that during coculture in the 3D3C chip, neurons and CMs form both physical and functional connections via axons, as proven by immunocytochemical staining and gene expression analysis and by evaluating the CM beating response during chemical stimulation of the neurons. The presented cardiac innervation on a chip is a valuable tool for studying the neuromuscular junctions between neurons and CMs in disease modeling, drug screening, and toxicity assays.

## 2. Results

### 2.1. Cardiac Innervation in the 3D3C Chip

The 3D3C chip consists of three serial compartments connected via microtunnels, allowing the culture of up to three different cell or tissue types in a single chip. Here, it was used in the coculture of hiPSC-neurons and -CMs, as the chip isolated neuronal somas in their own compartment, allowing axonal growth to the cardiac compartment through the microtunnels (SEM image of the microtunnels presented in Supplementary Figure S1A). Neurons were seeded into one side compartment, and axons traversed into the middle compartment for one week before seeding CMs. It has been shown that dendritic processes cannot traverse through microtunnels of 250-µm length [22], which was the length of microtunnels utilized in the chips here. Separate medium chambers for each compartment allowed culturing the cells in their respective media. The cocultures were evaluated at the 2- and 4-week timepoints, with a few being followed up to 8 weeks, showing good viability (data not shown). The 2- and 4-week timepoints in coculture were chosen based on previous data regarding neuronal activity. In microelectrode array (MEA) embedded cultures, neurons develop spontaneous network activity from two weeks onwards, activity peaking in ~4 weeks [18]. Here, at 2-week timepoint, the neurons have been cultured for 1 week alone and 2 weeks in coculture, and at 4-week timepoint for 1 week alone and 4 weeks in coculture.

**Figure 1:**
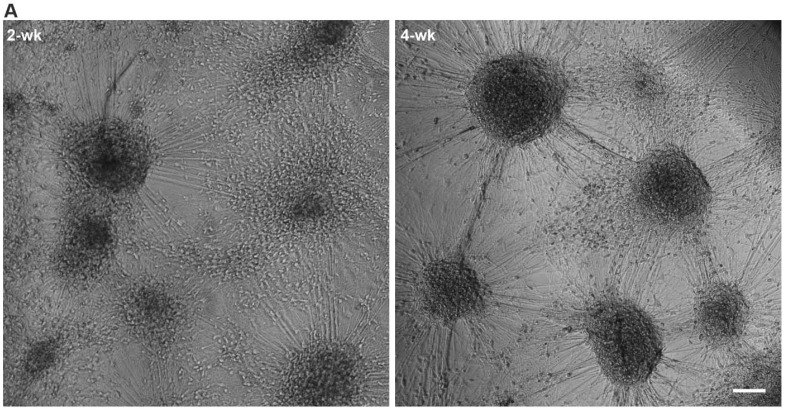

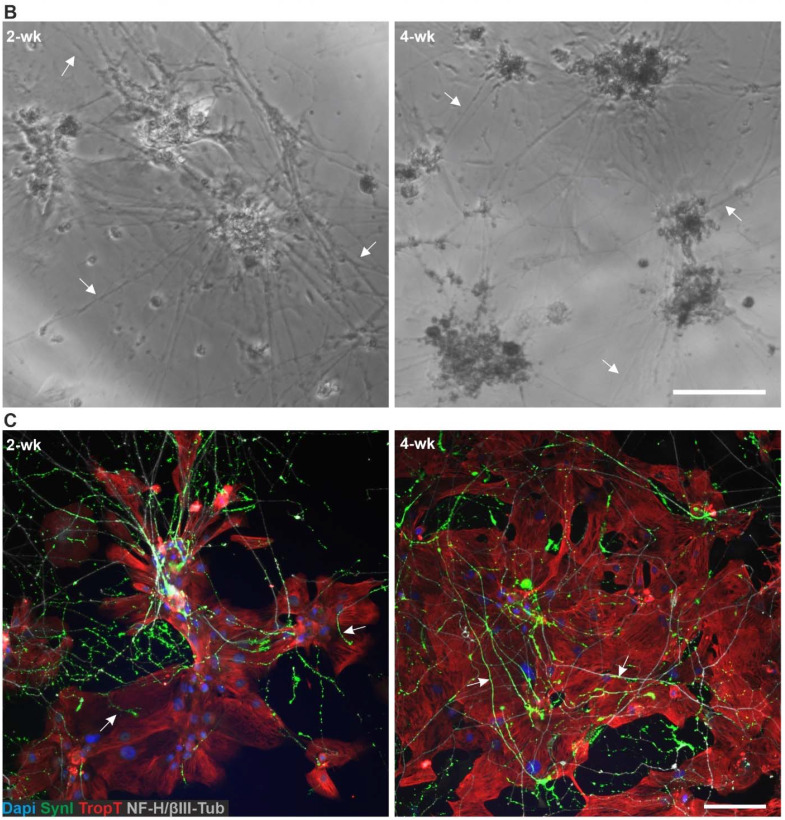
hiPSC-derived neurons and CMs in the 3D3C chip at 2- and 4-week timepoints of coculture. (**A**) A phase-contrast image showed viable neuronal somas isolated in their compartment and forming axonal networks. (**B**) Neuronal axons (white arrows) traversed to the middle compartment via microtunnels and CMs were viable among the axons. (**C**) ICC staining showed that neuronal axons stained against monoclonal anti-neurofilament NF-H 200 and monoclonal anti-β-tubulin III (NF-H/βIII-Tub, white) interacted with CMs stained against cardiac marker Troponin T (TropT, red) in the middle compartment. Evidence of synaptic interactions between CMs (TropT, red) and neurons (Synapsin I (SynI), green) can also be observed (white arrows). The scale bar is 100 µm, and blue indicates a nuclear marker (DAPI).

**Figure 2:**
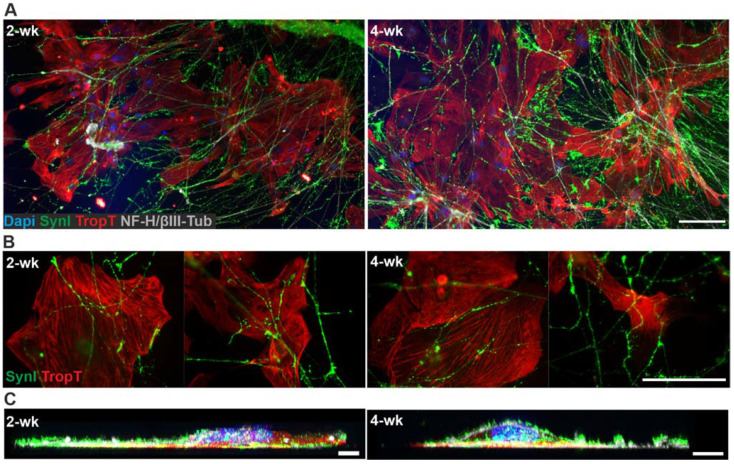
ICC staining of the hiPSC-derived neuron–CM coculture in the 3D3C chip at 2- and 4-week timepoints. (**A**) The staining showed the extension of the neuronal axons (NF-H/βIII-Tub, white) in the middle compartment among the CMs (TropT, red) and that both cell types were viable when in close contact with each other at both timepoints. (**B**) The staining of the synaptic marker (SynI, green) showed evidence of possible axon-mediated synaptic interactions with the CMs at both timepoints. The channels are shown separately in the Supplementary Figure S2. (**C**) 3D projection of the neuron–CM coculture showed that axonal synapses grew in close contact with CMs (NF-H/βIII-Tub, white; SynI, green; TropT, red). The scale bar is 100 µm for (**A**) and (**B**) and 10 µm for (**C**). Blue indicates a nuclear marker (DAPI).

Phase-contrast imaging of the 3D3C chips showed that neurons were viable and formed neuronal networks in their compartment (Figure 1A), and axons started to extend to the middle compartment through microtunnels immediately after seeding and elongated continuously throughout the experiments (Figure 1B, Supplementary Figure S1B). The CMs were viable among the axons (Figure 1B), and CM beating was observed during the whole coculture period (Supplementary Video S1). Immunocytochemical (ICC) staining showed that the monoclonal anti-neurofilament NF-H 200 and monoclonal anti-β-tubulin III (NF-H/βIII-Tub)-positive axons traversed through the microtunnels into the middle compartment and grew alongside the cardiac marker Troponin T (TropT)-positive CMs (Figure 1C and Figure 2, Supplementary Figure S1C). The synaptic marker Synapsin I (SynI) was expressed in the axons showing that the formation of functional connections between neurons and CMs was possible (Figure 2B, Supplementary Figure S2). 3D projections of the fluorescent confocal images showed that the axonal synapses grew in close contact with the CMs (Figure 2C, Supplementary Videos S2 and S3).

### 2.2. Gene Expression of hiPSC-Neurons and -CMs Cocultured in the 3D3C Chip

**Figure 3:**
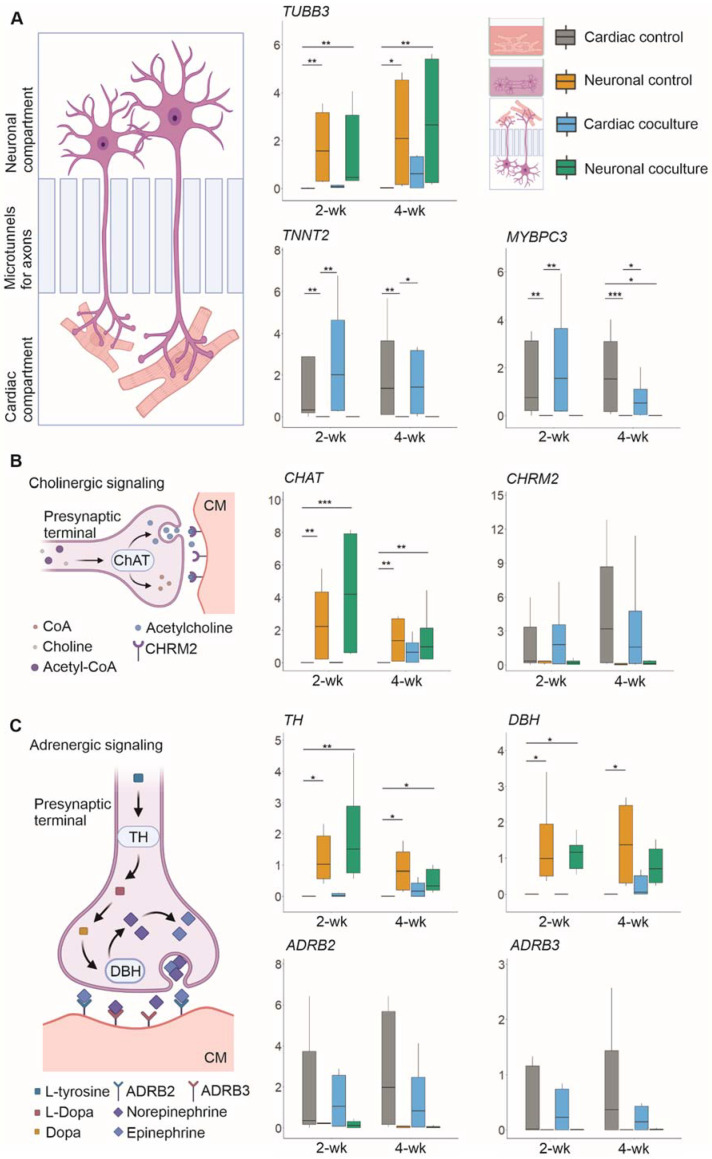
Relative expression of genes related to neuronal and cardiac identity, as well as cholinergic and adrenergic signaling. The data are presented as boxplots showing the median, upper, and lower quartiles and minimum and maximum values. (**A**) A schematic illustration of the 3D3C chip compartments. The expression of neuron-specific *TUBB3* was higher in all neuronal samples than in cardiac samples, whereas cardiac-specific *TNNT2* and *MYBPC3* expression was higher in all cardiac samples. (**B**) A schematic illustration of the cholinergic signaling pathway between neurons and CMs. The expression of *CHAT* was higher in neuronal samples, whereas the expression of *CHRM2* was higher in cardiac samples. (**C**) A schematic illustration of the adrenergic signaling pathway. *TH* and *DBH* were more highly expressed in neuronal samples, whereas *ADRB2* and *ADRB3* were more highly expressed in cardiac samples. The illustrations were created using BioRender.com. * *p* < 0.05, ** *p* < 0.01, *** *p* < 0.001.

The expressions of cardiac-specific genes *TNNT2* and *MYBPC3*, and neuron-specific gene *TUBB3* (Figure 3A) were evaluated to confirm that the samples from neuronal and cardiac compartments could be extracted without samples mixing. *TNNT2* and *MYBPC3* were expressed robustly in all cardiac samples, (2- and 4-week control samples, and 2- and 4-week coculture samples), whereas their expression in the neuronal samples was almost undetectable, with significant differences when compared to the cardiac samples. *TUBB3* was robustly expressed in neuronal samples, with significantly lower expression in cardiac samples. However, 4-week cardiac coculture samples had higher *TUBB3* expression than other cardiac samples suggesting an increase in the axonal expression of *TUBB3* in the coculture. It was shown earlier that *TUBB3* is detectable axon-specifically in neurons [23,24]. The detailed mean expressions and standard deviations, along with *p*-values for each gene and sample, are presented in Supplementary Table S1, and detailed information of the genes and TaqMan Assays used are presented in Supplementary Table S2.

The expression of the genes associated with the formation of the functional connection between neuronal axons and CMs was also evaluated. *CHRM2* and *CHAT* enable cholinergic signaling between neurons and CMs (Figure 3B), whereas *ADRB2*, *ADRB3*, *TH*, and *DBH* are related to adrenergic signaling (Figure 3C) [25]. *CHAT*, encoding choline O-acetyltransferase, was expressed mainly in the neuronal samples, with significant differences compared to the cardiac samples. However, *CHAT* expression in the cardiac coculture samples was higher compared to cardiac control samples, especially at the 4-week timepoint. The *CHRM2* gene, encoding muscarinic acetylcholine receptor M2, showed higher expression in the cardiac samples than the neuronal samples.

*TH* and *DBH* encode tyrosine hydroxylase and dopamine beta-hydroxylase, respectively. *TH* is involved in the conversion of L-tyrosine to L-dopamine, whereas *DBH* is involved in the conversion of dopamine to norepinephrine, which can be further converted into epinephrine [25]. Generally, *TH* and *DBH* were expressed more in the neuronal than cardiac samples, with significant differences except for 4-week cardiac coculture samples, which could be due to increased axonal expression of these genes. Two types of beta-adrenergic receptors are present in cardiomyocytes: *ADRB2* encodes a beta-2 adrenergic receptor with affinity for epinephrine [25], and *ADRB3* encodes a beta-3 adrenergic receptor with affinity for norepinephrine [25]. Their expressions were lower in the neuronal samples than in the cardiac samples.

### 2.3. Functional Connections Formed between hiPSC-Neurons and -CMs

Similar to 2D control samples, the hiPSC-CMs exhibited normal beating and contractile properties when cocultured with hiPSC-neurons in the 3D3C chip, and the elongated axons in the cardiac compartment did not disturb the video analysis (Supplementary Videos S1 and S4).

**Figure 4:**
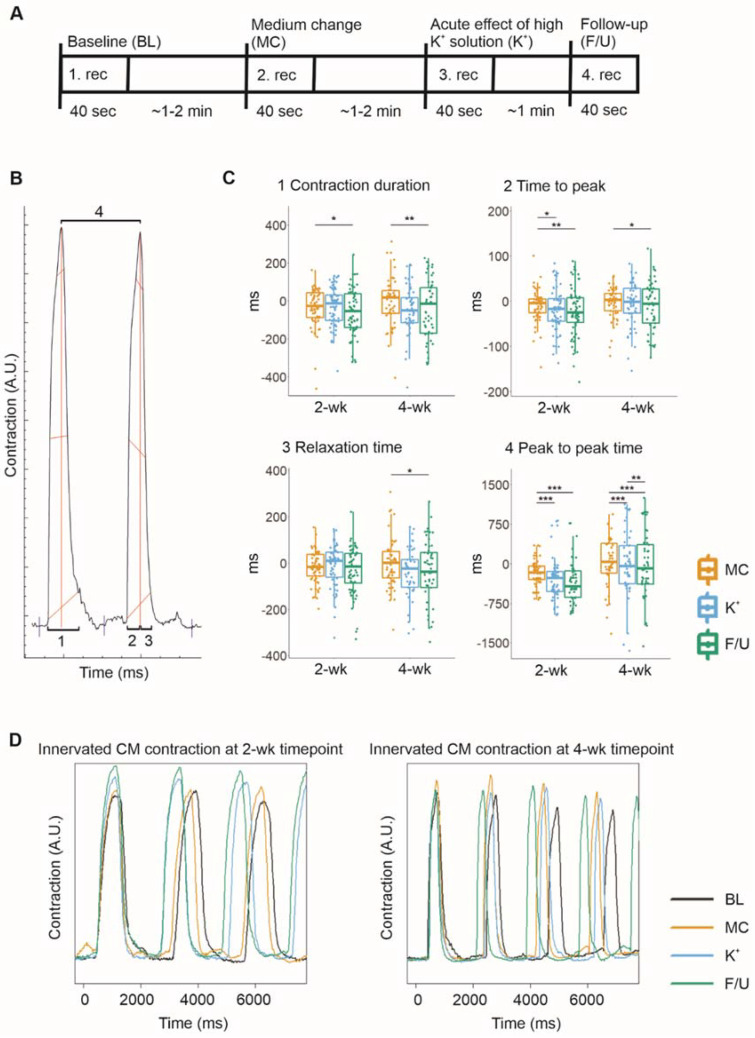
The evaluation of the functional connections between the cocultured hiPSC-neurons and hiPSC-CMs was performed by video microscopy recording of CMs beating during high K^+^ exposure of the neurons. The data are presented as boxplots overlaid by individual datapoints. (**A**) Experimental timeline for the recording of CMs beating during neuronal exposure to a high-K^+^ solution. (**B**) MUSCLEMOTION was used to analyze the videos for the contraction duration (1), time to peak (2), relaxation time (3), and peak-to-peak time (4). (**C**) The response to medium change (MC) was considered the baseline response, to which the immediate (K+) and follow-up (F/U) responses to neuronal high K^+^ exposure were compared. After K^+^ exposure, the contraction duration was observed to decrease, mostly due to a decrease in the time to peak. Furthermore, the peak-to-peak time of cardiomyocyte beating was observed to decrease, indicating an increased beating rate. (**D**) Examples of contraction curves acquired from MUSCLEMOTION analysis of the recorded videos. * *p* < 0.05, ** *p* < 0.01, *** *p* < 0.001.

Fluorescent ICC images showed that CMs grew in close contact with axons, which expressed synaptic proteins when in contact with CMs (Figure 2). Furthermore, qPCR results showed that both cell types expressed necessary genes for the formation of functional connections (Figure 3). However, these results only implied that functional connections were possible; therefore, the functionality of neuron–CM connections in the cocultures was evaluated at 2- and 4-week timepoints using video microscopy recording of CM beating during neuronal stimulation by a high-K^+^ solution. CM beating was recorded at baseline (1. rec), during medium change in the neuronal compartment (2. rec), during neuronal stimulation (3. rec) and at follow-up after approximately 2 min (4. rec) (Figure 4A). Videos were analyzed for contraction duration (10% above baseline), time to peak, relaxation time and peak-to-peak time using MUSCLEMOTION (Figure 4B). To observe the change in the CM beating and contractile properties during the neuronal stimulation, the baseline values for each evaluated parameter were subtracted from the medium change (MC), as well as the immediate (K^+^) and follow-up (F/U) of neuronal stimulation. The CM response to MC was considered as the baseline response and K^+^ and F/U responses were then compared to MC response.

The contraction duration indicates the time from the contraction start to the relaxation end and was observed to decrease during neuronal stimulation and follow-up at both timepoints with stronger at the 4-week timepoint (Figure 4C). The mean change in contraction duration when compared to baseline was −28.88 ± 122.77 for MC, −30.93 ± 102.98 for K^+^, and −52.63 ± 135.75 ms for F/U (*p* = 0.021 vs. MC) at the 2-week timepoint and −13.66 ± 195.20 for MC, −66.27 ± 214.63 for K^+^, and −90.61 ± 292.92 ms for F/U (*p* = 0.005 vs. MC) at the 4-week timepoint.

The time to peak and relaxation time were evaluated to determine whether neuronal stimulation affected CM contraction or relaxation. The time to peak indicates the time from the contraction start to the peak contraction. It decreased slightly during neuronal stimulation and follow-up at both timepoints (Figure 4C), the mean change being −12.49 ± 54.55 for MC, −14.76 ± 54.88 for K^+^ (*p* = 0.027 vs. MC), and −22.77 ± 54.40 ms for F/U (*p* = 0.004 vs. MC) at the 2-week timepoint and −3.88 ± 35.82 ms for MC, −8.33 ± 47.86 ms for K^+^, and −14.74 ± 60.11 ms for F/U (*p* = 0.031 vs. MC) at the 4-week timepoint. On the other hand, the relaxation time indicates the time from the peak contraction back to the relaxed state. It did not change significantly at the 2-week timepoint (Figure 4C) but decreased during neuronal stimulation and follow-up at 4-week timepoint (Figure 4C). The mean change was −16.24 ± 108.36 for MC, −15.95 ± 99.53 for K^+^, and −29.29 ± 111.30 ms for F/U at the 2-week timepoint, whereas it was −11.02 ± 177.55 for MC, −59.00 ± 194.04 for K^+^, and −77.56 ± 269.28 ms for F/U (*p* = 0.02 vs. MC) at the 4-week timepoint.

The peak-to-peak time, describing the CM beating rate, decreased significantly during high K^+^ exposure and follow-up at the 2-week and even more strongly at the 4-week timepoint, indicating an increased beating rate (Figure 4C). The mean change observed in the peak-to-peak time compared to baseline was −174.63 ± 242.31 for MC, −283.81 ± 411.11 for K^+^ (*p* = 0.00019 vs. MC), and −299.27 ± 483.76 ms for F/U (*p* = 0.00019 vs. MC) at the 2-week timepoint while it was 118.14 ± 841.95 for MC, −51.55 ± 1280.77 for K^+^ (*p* = 0.00090 vs. MC), and −181.07 ± 1426.18 ms for F/U (*p* = 0.00035 vs. MC and *p* = 0.004 vs. K^+^) at the 4-week timepoint.

## 3. Discussion

Here, we present an advanced, completely human cell-based, functional cardiac innervation on a chip in which hiPSC-neurons and -CMs were cocultured long-term in a compartmentalized 3D3C chip. Both cell types were seeded in their respective compartments, allowing only axonal interaction between the cell types. The system allowed us to study interactions between the two cell types under more in vivo-like conditions, where the interaction between cANS and cardiac tissue is restricted to occur between axons and CMs. Furthermore, the separate medium chambers allowed the coculture of cells in their respective media, eliminating the need to compromise between the medium needs of each cell type. The 3D3C chip allowed the usage of diverse cell analysis methods, including RNA extraction for gene expression studies, ICC for evaluating protein expression and cellular morphology, and functionality studies with video microscopy. Furthermore, the analyses can be performed separately for each chip compartment, thus individually for each cell type. The elegant chip design enables long-term studies, which are crucial for functional maturation and innervation formation.

The phase-contrast and ICC images showed that both cell types remained viable in the chip for 4 weeks and even up to 8 weeks. Furthermore, axon-specific NF-H and βIII-Tub and cardiac-specific TropT analyses showed that axons and CMs expressed the expected axonal and cardiac markers and their typical morphology, respectively. Axons expanded in the cardiac compartment throughout the experiments and created a visibly denser and longer network at the 4-week compared to the 2-week timepoint, providing more axonal connections for the CMs, and possibly enhancing the neuron–CM interaction. 3D projections of cocultures confirmed that axons and the clustered synaptic protein SynI were located very close to CMs, indicating that neurons and CMs can interact via axon-mediated synaptic connections. In previous neuron–CM coculture studies, the presence of synaptic proteins near CMs has not been evaluated except for by Takayama et al. [13], although later, they showed that hiPSC-neurons expressed synaptic proteins in free cocultures with CMs [15].

The RT-qPCR results showed that the hiPSC-neurons and -CMs expressed their cell-specific markers, *TUBB3* for neurons and *TNNT2* and *MYBPC3* for CMs, indicating the proper identity of the cells and that RNA can be extracted separately from the neuronal and cardiac compartments without samples mixing. This enabled the evaluation of gene expression separately for each cell type. Due to the low cell amount, RNA was pooled from three cell-specific compartments to form one sample. Although pooling RNA samples results in loss of information due to loss of sample variability, it is used in gene expression studies, e.g., for budgeting reasons or insufficient RNA input [26]. Gene expression has not been analyzed in previous publications regarding neuron–CM coculture in a chip [10,11,12,13] but is important in characterization of the cell types and their maturity, and a commonly used analysis method in organ and body on a chip studies [27,28].

We evaluated the expression of genes enabling cholinergic and adrenergic signaling between neurons and CMs. The expression of *CHAT* and *CHRM2* are required for cholinergic signaling; *CHAT* encodes protein involved in neurotransmitter acetylcholine conversion and *CHRM2* is acetylcholine receptor in CMs [25]. *CHAT* was expressed robustly in neuronal samples, whereas its expression in cardiac control samples was very low. The *CHAT* expression was higher in cardiac coculture samples, especially in the 4-week coculture, implying that the axonal *CHAT* expression increased temporally. Protein synthesis can occur locally in axons when mRNA translocates from the neuronal soma to the axonal regions [23,24,29]. The increase from the 2- to 4-week timepoint suggests a temporal increase in the growth and maturation of the axons. On the other hand, *CHRM2* expression was robust in cardiac samples, but low in neuronal samples. Regarding adrenergic signaling, *TH* and *DBH* were robustly expressed in the neuronal samples, whereas *ADRB2* and *ADRB3* showed higher expression in cardiac samples. Similar to *CHAT*, *TH* and *DBH* showed higher expression in 4-week cardiac coculture samples than in cardiac control samples, where their expression was almost absent, indicating increased axonal expression of these genes.

Together, the RT-qPCR results indicated that both cell types are capable of signaling via both cholinergic and adrenergic pathways, including neurons to synthesize their specific neurotransmitters, and CMs and their specific receptors to bind the neurotransmitters. The neurons expressed both cholinergic and adrenergic genes, suggesting that there are neurons with both sympathetic and parasympathetic characteristics. Although there has been an indication of the formation of functional connections between neurons and CMs when cocultured on chips in previous studies, they have not shown whether the two cell types express the genes or proteins necessary for the connections to form. Oiwa et al. showed that neurons cultured on their chip expressed *CHAT* and *DBH* at the protein level, but they did not evaluate the expression of cardiac receptors [11]. However, the earlier studies mostly used primary cells known to express these genes in vivo, even though harvesting the cells from their natural environment could affect the gene expression [30]. On the other hand, hiPSC-neurons and hiPSC-CMs differ from adult neurons and CMs in some respects, making it important to evaluate the capability of the cells to form functional connections [31,32].

Video microscopy analysis of CMs showed that the CMs exhibited normal beating characteristics when cocultured with the neurons in the chip. Importantly, the large video dataset collected and analyzed from CMs during high K^+^ exposure to the neuronal compartment indicated that the axon-mediated connections between the neurons and CMs were electrophysiologically functional already at 2-week timepoint and became stronger at 4-week timepoint. When the neurons were exposed to high-K^+^ solution, significant changes in the CM beating characteristics were observed in both the 2- and 4-week timepoints, including an increase in the beating frequency and a decrease in the contraction duration. The chip compartments have been previously shown to be fluidically isolated [33], indicating that the changes in cardiomyocyte beating is due to the neuronal stimulation and not due to potassium leakage through the microtunnels. This observation indicated that the activation of adrenergic signaling in neurons, as the CM response to neuronal stimuli was excitatory. The adrenergic signaling pathway is known to increase heart rate and strengthen the contractility of cardiomyocytes, whereas the cholinergic signaling pathway decreases heart rate [34].

Previously, the functionality of neuron–CM interactions has been reported in models utilizing rat primary neurons and CMs [10,11] and rat primary neurons and hiPSC-CMs [12] with integrated MEAs. Furthermore, MEAs have been utilized in cardiac innervation models where CMs have been seeded on top of neurons [15,35]. In these studies, neuronal stimulation evoked a response in the CMs. Depending on whether the neurons used were sympathetic or parasympathetic, an increase or decrease in cardiac beating frequency, respectively, was observed [10,11,12,15,35]. Furthermore, Oh et al. and Winbo et al. used video microscopy analysis for their unrestricted cocultures to evaluate the functionality of the neuron–CM interactions; however, they reported only cardiac beating frequency [14,35]. In the present study, we observed changes in several contractile parameters, including the contraction duration, and time to peak and relaxation time, when stimulating hiPSC-neurons during coculture in the 3D3C chip.

The present study has aspects to be developed further in the future. Here, we used neurons originally pruned towards cortical phenotypes [18]. However, according to the gene expression and functional responses, neuronal population contained characteristics of both sympathetic and parasympathetic neurons, thus most likely they are able to adopt their phenotype according to the contact they made with cardiomyocytes. Furthermore, as the cardiomyocyte response to neuronal stimulation was observed, we have shown here that the cell types used form a functional connection. There are several protocols for differentiating specific types of neurons [15,36,37], which can be utilized in our future studies of the neuron–CM interaction and further evaluate the CM response to specific peripheral types of neurons. Moreover, we used neuronal high K^+^ exposure and video microscopy to evaluate the functionality of the neuron–CM interactions, although previous studies [10,11,12] have used MEAs to electrically stimulate neurons and record the CM response, allowing better spatiotemporal induction of the stimulation and the measurement of the response in both neurons and CMs. However, high K+ stimulation of hiPSC-neurons was previously characterized [38], and video analysis of the hiPSC-CM response to neuronal stimulation allows the evaluation of only the cardiac response, without axonal field potentials affecting the analysis. Furthermore, video analysis allows the extraction of multiple parameters of individual beating cardiomyocytes, which would require a single electrode per CM in chip models, not yet available. In the future, the 3D3C model should be further developed to include transparent MEAs [39] to facilitate both MEAs and video recording analyses of neuron–CM interactions.

We presented an advanced, electrophysiologically functional, and completely human-based cardiac innervation on a chip. The hiPSC-neurons and -CMs were cocultured with their respective media in separate compartments connected via microtunnels, enabling axonal growth from the neuronal to the cardiac compartment. The 3D3C chip enabled the use of several analysis methods to evaluate the functionality of the axon–CM connections. By combining immunocytochemical, gene expression and video recording analyses, we showed the formation of physical connections between the cell types that required signaling machinery for synaptic activation of CMs, which was further verified with a functional response to neuronal stimulation. The chip supported long-term cocultures, and functional innervation was detected at 2 weeks, with enhanced maturation at 4 weeks. The 3D3C cardiac innervation on a chip is a valuable tool for screening drug adverse effects on neurons or CMs via the functional connections between them, as well as for studying the development of neuromuscular junctions and comaturation of the two cell types.

## 4. Materials and Methods

### 4.1. Cell Lines

hiPSC line 10212.EURCCS [40] was used for neuronal differentiation towards cortical neurons, and the hiPSC line UTA.04602.WT [41] was used for cardiac differentiation. The hiPSCs used were acquired from voluntary subjects who had given written and informed consent. The institute has a supportive statement from Pirkanmaa Hospital District to generate IPSCs from donor cells (R08070 and R12123) and to use generated cell lines in neuronal research (R05116). The pluripotency of the lines was confirmed regularly, and all cultures maintained normal karyotypes and were free of mycoplasma.

### 4.2. Neuronal Differentiation

Before neuronal differentiation, hiPSCs were transferred to feeder-free culture on 15 μg/mL of human recombinant laminin-521 (LN521, BioLamina, Sundbyberg, Sweden) using E8 medium (Thermo Fisher Scientific, Waltham, MA, USA) as previously described [42]. Neuronal differentiation included neuronal induction, precursor expansion and maturation phases and was performed as previously described [18]. On day 32 of differentiation, cells were seeded for experiments in neural maturation medium consisting of a 1:1 mixture of D-MEM/F12 (with GlutaMAX) and neurobasal medium, 0.5% N2, 1% B27 with retinoic acid, 0.5 mM of GlutaMAX, 0.5% NEEA, 50 μM of 2-mercaptoethanol, 0.1% penicillin/streptomycin (all from Thermo Fisher Scientific), 2.5 μg/mL of insulin (Sigma-Aldrich, Saint Louis, MO, USA), 20 ng/mL of brain-derived neurotrophic factor (BDNF, R&D Systems, Minneapolis, MN, USA), 10 ng/mL of glial-derived neurotrophic factor (GDNF, R&D Systems), 500 μM of dibutyryl-cyclic AMP (db-cAMP, Sigma-Aldrich), and 200 μM of ascorbic acid (AA, Sigma-Aldrich). During cell detachment on day 32, 10 μM ROCK inhibitor (Sigma-Aldrich) was added to the medium to support cell survival.

### 4.3. Cardiac Differentiation

Before cardiac differentiation, hiPSCs were expanded on mouse embryonic fibroblasts (CellSystems GmbH, Troisdorf, Germany) in KSR medium (KnockOut DMEM, Gibco, Waltham, MA, USA) containing 10% KnockOut Serum Replacement (Gibco), 1% MEM NEAA (Gibco), 1% GlutaMAX, 0.2% β-mercaptoethanol (Gibco), and 0.5% penicillin/streptomycin (Lonza, Basel, Switzerland). Embryoid body (EB) differentiation was performed as recently described [20]. On day 21 of differentiation, CMs were isolated from other cell types using magnetic-activated cell sorting (MACS). The MultiTissue Dissociation Kit (Miltenyi Biotec, Bergisch Gladbach, Germany) was used to dissociate the EBs, and PSC-Derived Cardiomyocyte Isolation Kit, human (Miltenyi Biotec), was used to isolate CMs as described previously [31]. Isolated CMs were suspended in 20% EB medium (KnockOut DMEM containing 20% FBS (Gibco), 1% MEM NEAA, 1% GlutaMAX, and 0.5% penicillin/streptomycin).

### 4.4. Preparation of the 3D3C Chips and Coculture

#### 4.4.1. Chip Manufacturing

**Figure 5:**
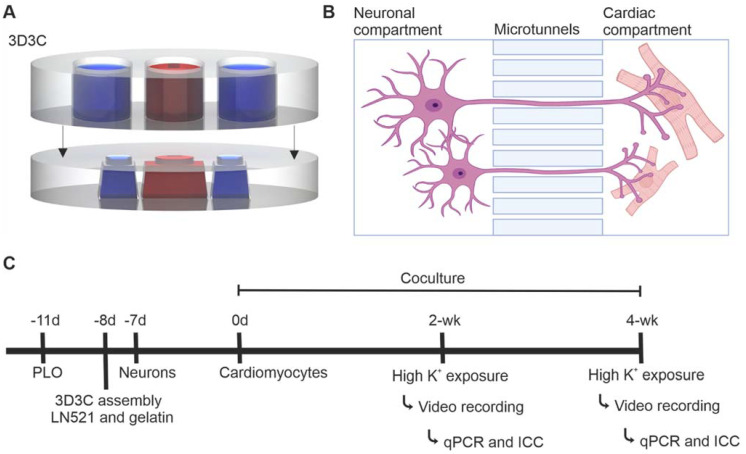
The compartmentalized microfluidic device (3D3C chip) and the timeline of the experiment. (**A**) A schematic illustration of the 3D3C chip containing two PDMS-based parts: the cell culturing part (lower) and the medium chamber part (upper). The cell culturing part had three separate compartments for the cells, and the medium chamber part had three separate chambers for each cell culture compartment. (**B**) A schematic illustration of the microtunnels between two cell culture compartments, allowing axonal growth into the adjacent compartment. Neurons and CMs can be cultured in their own cell compartments with their respective media. The illustration was created with BioRender.com. (**C**) The experimental design of the study. Neurons and CMs were cocultured for 2- or 4-week period, after which video microscopy analysis, immunocytochemical staining and RT-qPCR were performed.

The compartmentalized PDMS-based microfluidic device, called the 3D3C chip, originally designed for axonal isolation studies [21,22], was used to obtain neuronal soma isolation (Figure 5A). The 3D3C chip consists of two PDMS-based parts, the cell culturing part attached on glass coverslip and the medium chamber part attached on top of the cell culturing part. The cell culturing part of the chip consists of two neuronal compartments on both sides (length = 3, width = 4 mm) and a coculture compartment in the middle (length = 5, width = 4 mm). The three compartments are connected in series by 40 microtunnels (length = 250, width = 10, height = 3.5 μm) (Figure 5A,B). The medium chamber part attached to the cell culture part consisted of three separate chambers to enable the use of cell-specific medium for each cell type.

Both parts were produced from PDMS of 10:1 base and curing agent ratio (SYLGARD 184, Dow Corning, Midland, MI, USA) using molds fabricated with the previously described combination of SU-8 photolithography and 3D printing [21,22]. The medium chambers were cut from a 4 mm thick PDMS sheet with Epilog Laser Fusion 120 W (Epilog Laser, Golden, CO, USA), 7% speed, 70% power, and 70% frequency. The larger debris was removed with pressurized air, and the medium chambers were washed using warm (+45 °C) distilled water containing dish soap and rinsed with distilled water. The medium chambers were placed in isopropanol-filled bubble bags (BuBclean, Enschede, The Netherlands) in a sonicator for 30 min, and isopropanol was replaced every 10 min, after which they were rinsed with distilled water, dried with pressurized air and cleaned with an acetone wipe. Thereafter, 5 min of sonication in isopropanol followed by distilled water rinsing and acetone wiping was repeated at least two times to remove any remaining residue from laser cutting. The parts were air-dried overnight. The 3D3C chips were imaged using scanning electron microscopy (SEM). The UltraPlus Scanning Electron Microscope (Zeiss, Oberkochen, Germany) was used for the imaging, and the 3D3C chip samples were prepared for the imaging by sputter-coating them with 4.5-nm-thick Pt/Pd.

#### 4.4.2. Preparation of the Coculture in the 3D3C Chip

The study design is shown in Figure 5C and the detailed number of samples for each experiment and sample type are presented in Supplementary Table S3. The preparation of 3D3C chips was performed as described earlier [22]. HCl-washed glass coverslips (22 × 22 mm) were coated with 0.25-mg/mL poly-L-ornithine (PLO, Sigma-Aldrich) for 1.5 h at +37 °C. Thereafter, coverslips were washed three times with sterile H_2_O, air-dried and stored at +4 °C. Polyvinylpyrrolidone (PVP)-treated 3D3C chips were immersed in 70% ethanol for sterilization and air-dried at RT. The 3D3C chips were assembled by manually attaching them on top of PLO-coated coverslips. The side cell culture compartments were coated with 30 µg/mL LN521, whereas the middle compartment was coated with 0.1% gelatin (Type A porcine gelatin, Sigma-Aldrich). After overnight incubation at +4 °C, the chips were ready to use.

Forty thousand neurons were seeded in one of the side compartments at day 32 of differentiation. Neurons were cultured alone for one week using neuronal medium in all chip compartments, after which MACS-sorted CMs were seeded in the middle compartment. A total of 3000–4000 cells produced the best CM sheets without extensive growth of unwanted cell types over the 2- or 4-week coculture. The neuronal medium was changed to both neuronal and empty side compartments whereas EB medium was changed to middle cardiac compartment three times a week. Very little medium diffusion occurred via the axonal microtunnels connecting the compartments (Figure 5A,B) [33].

Plastic and MatTek 48-well plate control samples were prepared following the same coating protocol except using 0.1 mg/mL PLO for 1 h and 15 µg/mL LN521. Wells for neurons and neuron–CM cocultures were coated with LN521, whereas wells for CMs were coated with gelatin. Neuron–CM cocultures were cultured in 1:1 neuronal and EB medium, while neuronal and cardiac monocultures were cultured in their respective media.

### 4.5. Analysis Methods of the Coculture in the 3D3C Chip

#### 4.5.1. RT-qPCR

Samples were collected, and RNA was extracted with a NucleoSpin RNA kit (Macherey-Nagel, Düren, Germany) following the manufacturer’s instructions immediately after video microscopy recording of CMs and chemical stimulation of neurons. Samples were lysed in buffer RA1, containing 3.5 µL of b-mercaptoethanol separately from the neuronal and cardiac compartments, and the content of three cell-specific compartments of the 3D3C chips were pooled to make one sample. Control samples in plastic well plates were lysed similarly, with one well containing one sample. RNA was extracted from the cell lysate and eluted in 20 µL of RNase-free H_2_O provided in the kit, after which the RNA quality and concentration were measured using a NanoDrop (Thermo Scientific).

Reverse transcription was performed with a High-Capacity cDNA Reverse Transcription kit (Applied Biosystem, Waltham, MA, USA) following the manufacturer’s instructions. Briefly, 10 µL of prepared 2× RT Master Mix and 10 µL of RNA sample were mixed. In some cases, RNA samples were diluted in nuclease-free water to even the RNA concentration in the reactions. Samples were then run in Mastercycler EP Gradient S (Eppendorf, Hamburg, Germany) using the following protocol: 10 min at +25 °C, 120 min at +37 °C, 5 min at +85 °C, and +4 °C until storage at −20 °C.

cDNA preamplification was performed due to the low RNA concentration of the 3D3C chip samples by using 2× TaqMan PreAmp Master Mix (Applied Biosystem) following the manufacturer’s instructions. The TaqMan 20× assays used are listed in Supplementary Table S2 and were tested with commercial Human Fetal Brain Total RNA (Takara Bio, Kusatsu, Japan) and Total RNA Normal Human Heart (Amsbio, Cambridge, MA, USA). Briefly, a 0.2× TaqMan Assay Pool was prepared using TaqMan 20× Assays for *ADRB2*, *ADRB3*, *CHRM2*, *MYBPC3*, *TNNT2*, *CHAT*, *TH*, *DBH*, *TUBB3*, *GAPDH*, *EEF1A1*; *EE+*, and *GUSB*. Reactions were prepared by mixing 25 µL of TaqMan PreAmp Master Mix, 12.5 µL of 0.2× TaqMan Assay Pool and 12.5 µL of the cDNA sample. Samples were run in Mastercycler EP Gradient S using the following protocol: 10 min at +95 °C, 14 cycles of 15 s at +95 °C, and 4 min at +60 °C, 10 min at +99 °C, and at +4 °C until storage at −20 °C.

qPCR for preamplified cDNA samples was performed by using TaqMan Fast Advanced Master Mix (Applied Biosystem) following the manufacturer’s instructions. The same TaqMan 20× assays were used as in the preamplification step. Briefly, master mixes for each gene were prepared as instructed in the kit. Preamplified cDNA samples were diluted to nuclease-free water 1:5 (20 µL of sample in 80 µL of water). Two µL of the diluted sample was added to 6 µL of the master mix, and three technical replicates were used for each sample for each gene. The plates were run in a CFX384 Touch Real-Time PCR System (Bio-Rad, Hercules, CA, USA) with the following protocol: 2 min at +50 °C, 20 s at +95 °C, and 40 cycles of 3 s at +95 °C, and 30 s at +60 °C. The 2^−^^ΔΔ^^Ct^ method [43] was used to calculate the relative expression of the evaluated genes. *GAPDH*, *EEF1A1*; *EE+* and *GUSB* were used as endogenous controls. For *ADRB2*, *ADRB3*, *CHRM2*, *MYBPC3*, and *TNNT2*, a 2-week cardiac control was used for normalization, whereas for *CHAT*, *TH*, *DB*, and *TUBB3*, a 2-week neuron control was used for normalization.

#### 4.5.2. ICC and Imaging

Immunocytochemistry (ICC) was performed at 2 and 4 weeks of coculture immediately after video microscopy recording of cardiomyocytes and chemical stimulation of neurons. The ICC protocol was used for 3D3C chip samples as described earlier [22], with the modification of washing samples with 4′,6-diamidino-2-phenylindole (DAPI, 1:5000) diluted in PBS after secondary antibody incubation. The primary antibodies used included monoclonal anti-neurofilament NF-H 200 (NF-H, 1:500, mouse IgG1, N5389, Sigma-Aldrich), monoclonal anti-β-tubulin III (βIII-Tub, 1:500, mouse IgG2b, T8660, Sigma-Aldrich), anti-synapsin I (SynI, 1:500, rabbit IgG, 574777, Merck, Darmstadt, Germany) and anti-troponin T (TropT, 1:750, goat IgG, ab64623, Abcam, Cambridge, UK). The secondary antibodies used included Alexa Fluor 488 (1:200, donkey anti-rabbit, A21206), Alexa Fluor 568 (1:200, donkey anti-goat, A11057) and Alexa Fluor 647 (1:125, donkey anti-mouse, A31571, all from Thermo Fisher Scientific).

Forty-eight MatTek control samples were stained as previously described [18]. The dilutions of primary antibodies were 1:1000 for NF-H, βIII-Tub and SynI and 1:1500 for TropT. The dilutions of the secondary antibodies were 1:400 for Alexa Fluor A488 and A568 and 1:200 for Alexa Fluor A647. All the samples were mounted with Vectashield including DAPI (Antifade Mounting medium with DAPI, H-1200, Vector Laboratories, Burlingame, CA, USA). The staining was visualized with an IX51 inverted fluorescence microscope (Olympus, Tokyo, Japan) and an LSM 780 laser scanning confocal microscope (Zeiss). Confocal images were deconvoluted with Hyugens and processed with Imaris (Oxford Instruments, Abingdon, UK).

#### 4.5.3. Cardiac Video Recording and Analysis

Neurons were stimulated through the addition of a high-K^+^ solution to the neuronal compartment. The extracellular solution contained 93 mM of NaCl, 10 mM of 4-(2-hydroxyethyl)-1-piperazineethanesulfonic acid (HEPES), 10 mM of D-glucose, 50 mM of KCl, 1.25 mM of NaH_2_PO_4_, 2 mM of CaCl_2_, and 1 mM of MgCl_2_ (pH adjusted to 7.4 with NaOH). CM activity was recorded before neuronal stimulation for baseline measurement (1. Rec), during medium change in the neuronal compartment to evaluate the effect of the liquid change on CM beating (2. Rec), during neuronal high K^+^ exposure to capture the immediate CM response (3. Rec) and approximately 2 min after neuronal stimulation as a follow-up (4. Rec). All videos of CM activity were recorded for 40 s at 44 fps using an Eclipse TS100 microscope (Nikon, Tokyo, Japan) and IGV-B1620M-KC000 camera (Imperx Incorporated, Boca Raton, FL, USA). MUSCLEMOTION (version 1.0) software [44] was used to extract CM beating characteristics from the videos. The data were further analyzed with R (version 4.0.2, R Foundation for Statistical Computing, Vienna, Austria) [45] using in-house-developed scripts. As a control, the high-K^+^ solution was added directly to CMs, which caused the CMs to cease beating (Supplementary Videos S5–S8).

#### 4.5.4. Statistical Analysis

Statistical analysis was performed using R and the *rstatix* (version 0.7.0) package [46]. A nonparametric Kruskal–Wallis rank sum test followed by Dunn’s post hoc test with Holm correction were used for independent samples from the qPCR analysis (6 samples for each condition) grouped by timepoint (2- or 4-week). A nonparametric Wilcoxon signed rank test was used for paired samples from video analysis (65 regions of interest from 27 chips at 2-week timepoint and 53 regions of interest from 19 chips at 4-week timepoint). A significance level of *p* < 0.05 was considered statistically significant. The data are presented as the mean ± standard deviation in the text and as boxplots and boxplots with individual data points in the figures.

## Data Availability

All data are available from the authors by request.

## Supplementary Materials

The following supporting information can be downloaded at: https://www.mdpi.com/article/10.3390/ijms23063148/s1.
