# Supplementary material for: Human Neurons Form Axon-Mediated Functional Connections with Human Cardiomyocytes in Compartmentalized Microfluidic Chip
Source: Int J Mol Sci. 2022 Mar 15;23(6):3148. doi: 10.3390/ijms23063148 (PMC8955890; doi:10.3390/ijms23063148)
Supplement: Supplementary file 1 [file ijms-23-03148-s001.zip › Supplementary Figures S1-S2.pdf]

Supplementary Figures

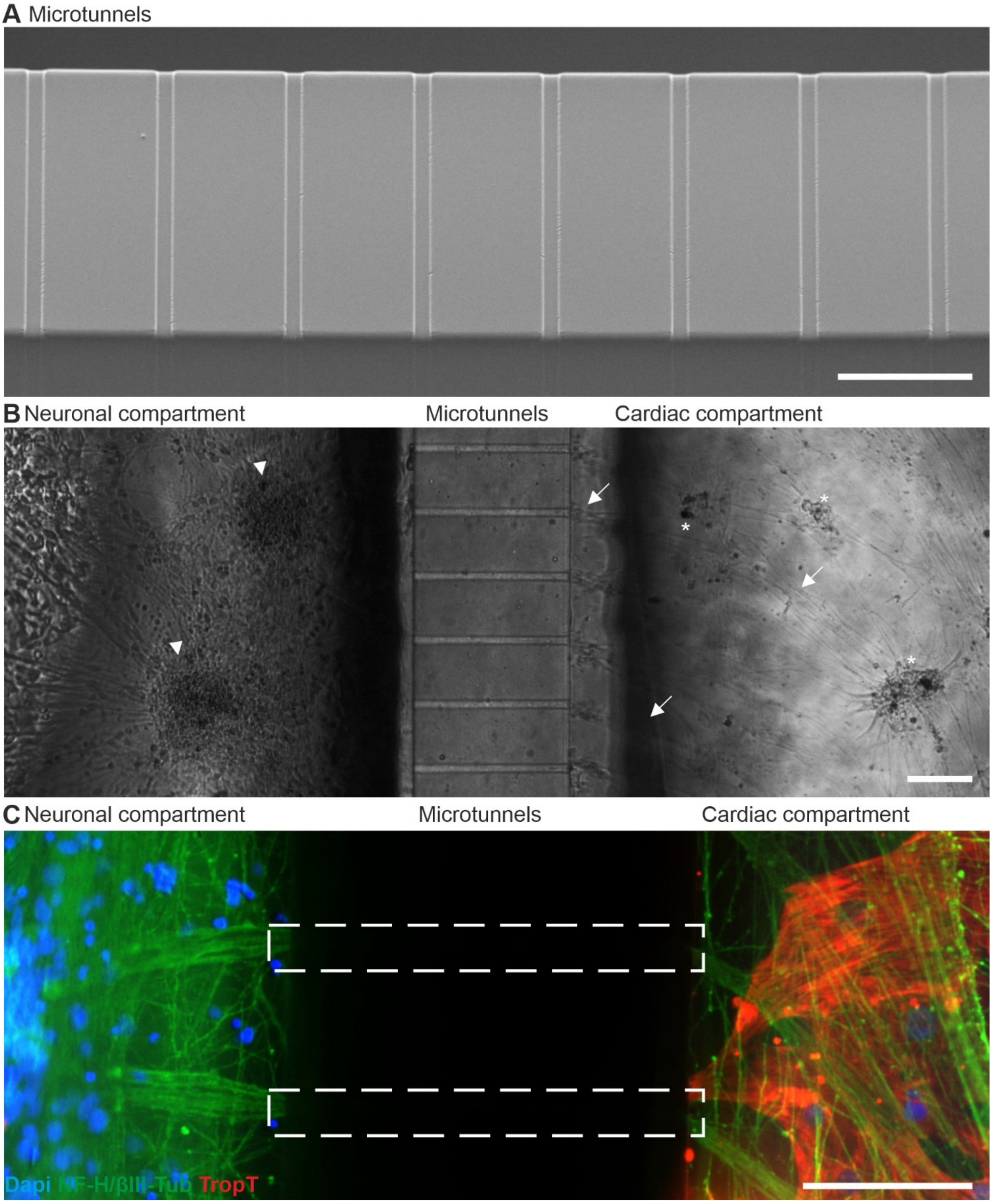

Supplementary Figure S1. The 3D3C chip microtunnels in SEM image and during coculture (scale bars 100 µm). A) SEM image of microtunnels in 3D3C chip. B) Phase contrast image showing neurons forming neuronal network (arrowheads) in their respective compartment and axons (arrows) traversing through microtunnels from neuronal compartment to cardiac compartment among CMs (asterisks). C) ICC image showing axons (NF-H/βIII-Tub, green) traversing through the microtunnels from neuronal compartment to cardiac compartment among CMs (TropT, red).

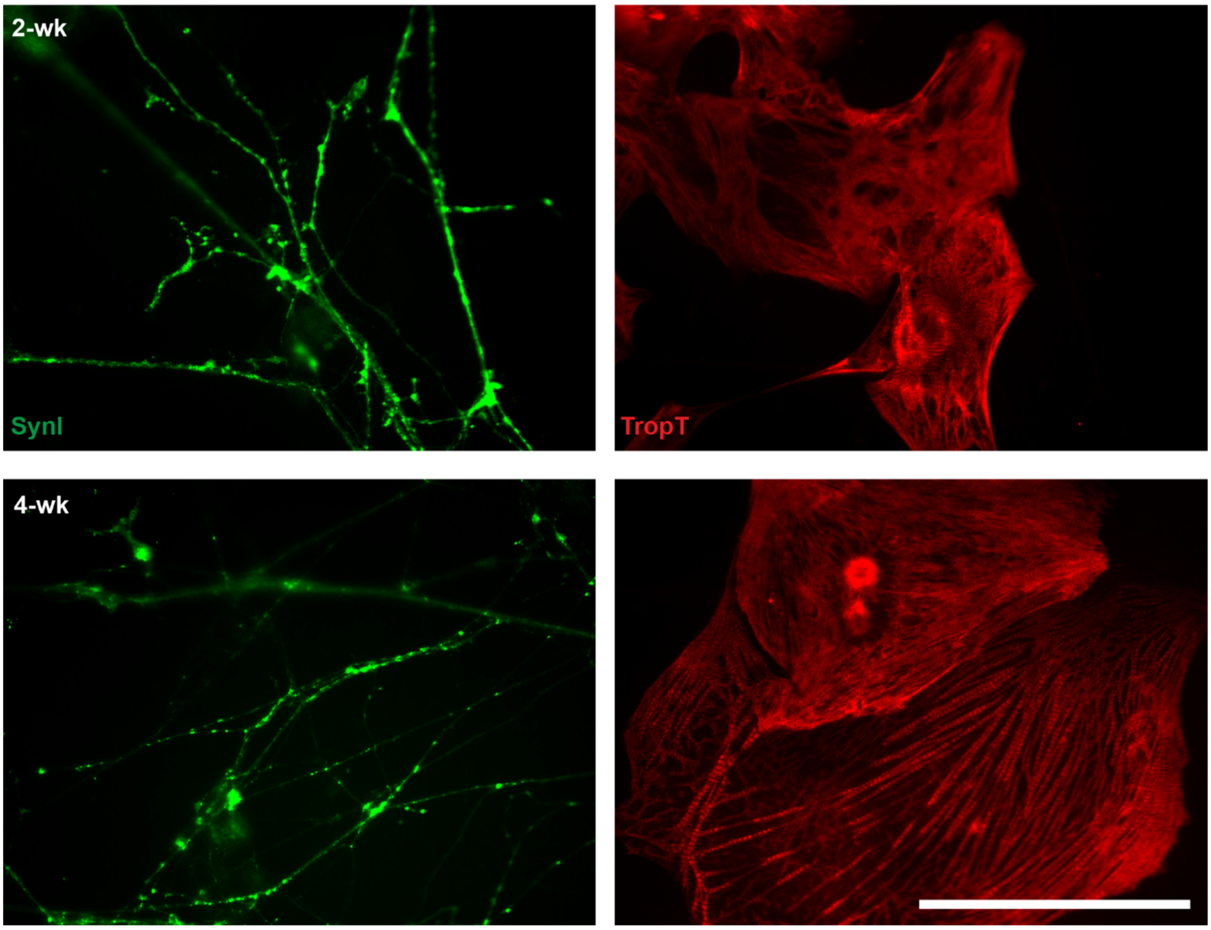

Supplementary Figure S2. Individual channels for Syn I and Trop T for Figure 2 (scale bar 100 μm).
