# Supplementary material for: Human Neurons Form Axon-Mediated Functional Connections with Human Cardiomyocytes in Compartmentalized Microfluidic Chip
Source: Int J Mol Sci. 2022 Mar 15;23(6):3148. doi: 10.3390/ijms23063148 (PMC8955890; doi:10.3390/ijms23063148)
Supplement: Supplementary file 1 [file ijms-23-03148-s001.zip › Supplementary Table S1_Relative expression of the genes evaluated from control and coculture samples.pdf]

Supplementary Table S1. Relative expression of the genes evaluated from neuronal control (N C), neuronal coculture (N CC), cardiac control (C C) and cardiac coculture (C CC) samples at 2- and 4-wk timepoints. The expressions are presented as mean ± standard deviation.

Sample	***TNNT2***	***MYBPC3***	***TUBB3***	***CHAT***	***CHRM2***	***TH***	***DBH***	***ADRB2***	***ADRB3***
2-wk N C	9.3E-6 ± 9.5E-6<br><br>*P*=0.0047 (2-wk C C)<br><br>*P*=0.0027 (2-wk C CC)	2.4E-5 ± 2.1E-5<br><br>*P*=0.0042 (2-wk C C)<br><br>*P*=0.0047 (2-wk C CC)	1.8 ± 1.6	2.5 ± 2.6	0.3 ± 0.2	1.2 ± 0.8	1.4 ± 1.2	0.2 ± 0.1	0.002 ± 0.002
4-wk N C	2.2E-5 ± 2.6E-5<br><br>*P*=0.0027 (4-wk C C and C CC)	3.8E-5 ± 3.8E-5<br><br>*P*=0.00088 (4-wk C C)	2.3 ± 2.4	1.4 ± 1.4	0.2 ± 0.3	0.9 ± 0.7	1.4 ± 1.2	0.1 ± 0.2	0.001 ± 0.002
2-wk N CC	0.04 ± 0.09	0.05 ± 0.1	1.6 ± 1.9	4.3 ± 4.0	0.3 ± 0.2	2.0 ± 1.6	1.1 ± 0.5	0.2 ± 0.2	0.01 ± 0.01
4-wk N CC	0.03 ± 0.04	0.008 ± 0.01<br><br>*P*=0.025 (2-wk C C)	2.8 ± 2.8	1.5 ± 1.7	0.2 ± 0.2	0.7 ± 0.7	0.8 ± 0.6	0.05 ± 0.05	0.01 ± 0.02
2-wk C C	3.4 ± 5.2	1.8 ± 1.6	0.02 ± 0.02<br><br>*P*=0.0063 (2-wk N C)<br><br>*P*=0.0036 (2-wk N CC)	0 ± 0<br><br>*P*=0.0097 (2-wk N C)<br><br>*P*=0.00073 (2-wk N CC)	2.2 ± 2.4	0.002 ± 0.004<br><br>*P*=0.011 (2-wk N C)<br><br>*P*=0.0026 (2-wk N CC)	0.02 ± 0.05<br><br>*P*=0.031 (2-wk N C)<br><br>*P*=0.022 (2-wk N CC)	2.4 ± 2.6	1.0 ± 1.5
4-wk C C	2.1 ± 2.4	1.8 ± 1.8	0.02 ± 0.02<br><br>*P*=0.024 (4-wk N C)<br><br>*P*=0.0036 (4-wk N CC)	6.9E-4 ± 1.7E-3<br><br>*P*=0.0092 (4-wk N C)<br><br>*P*=0.0054 (4-wk N CC)	4.8 ± 5.5	0.004 ± 0.002<br><br>*P*=0.015 (4-wk N C<br><br>*P*=0.024 (4-wk N CC)	0.1 ± 0.4<br><br>*P*=0.043 (4-wk N C)	2.8 ± 3.1	0.8 ± 1.1
2-wk C CC	2.7 ± 2.8	2.2 ± 2.4	0.3 ± 0.5	0.4 ± 1.0	2.5 ± 2.9	0.2 ± 0.5	0.7 ± 1.8	1.3 ± 1.4	0.6 ± 0.8
4-wk C CC	3.2 ± 5.0	0.7 ± 0.8	1.2 ± 1.7	0.7 ± 0.8	3.3 ± 4.5	0.2 ± 0.3	1.7 ± 3.7	1.4 ± 1.7	0.4 ± 0.5
