# Supplementary material for: Human Neurons Form Axon-Mediated Functional Connections with Human Cardiomyocytes in Compartmentalized Microfluidic Chip
Source: Int J Mol Sci. 2022 Mar 15;23(6):3148. doi: 10.3390/ijms23063148 (PMC8955890; doi:10.3390/ijms23063148)
Supplement: Supplementary file 1 [file ijms-23-03148-s001.zip › Supplementary Table S2_Number of samples for each analysis method and sample type.pdf]

Supplementary Table S2. The number of 3D3C chips and controls used in experiments. The experiment was repeated twice, and the table shows the total number of samples used. After video microscopy recordings of CMs and chemical stimulation of neurons, the same 3D3C chips were used in ICC and RT-qPCR analysis. In total, 68 pieces of 3D3C chips were utilized, of which 8 were excluded. Thus, a total of 60 chips were included in the ICC and RT-PCR analyses, among which 14 were excluded from chemical stimulation and video microscopy analysis.

Sample	***Chemical stimulus/ Video microscopy***	***ICC***	***RT-qPCR***
3D3C chip: 2-wk coculture	27 (n_{ROI} = 65)	12	18
3D3C chip: 4-wk coculture	19 (n_{ROI} = 53)	12	18
Control 48-wp: 2-wk culture	-	6 wells of each	6 wells of each
Control 48-wp: 4-wk culture	-	6 wells of each	6 wells of each
Direct chemical stimulus of CMs: 2-wk culture (Supplemental Information)	2 wells	-	-
Direct chemical stimulus of CMs: 4-wk culture (Supplemental Information)	2 wells	-	-
