# Supplementary material for: Human Neurons Form Axon-Mediated Functional Connections with Human Cardiomyocytes in Compartmentalized Microfluidic Chip
Source: Int J Mol Sci. 2022 Mar 15;23(6):3148. doi: 10.3390/ijms23063148 (PMC8955890; doi:10.3390/ijms23063148)
Supplement: Supplementary file 1 [file ijms-23-03148-s001.zip › Supplementary Table S3_20í┴ TaqMan Assays used in RT-qPCR.pdf]

Supplementary Table S3. 20x TaqMan Assays used in RT-qPCR.

Gene	***Description***	***Function***	***TaqMan 20X Assay ID***
ADRB2	Beta-2 adrenergic receptor	Adrenergic receptor	Hs00240532_s1
ADRB3	Beta-3 adrenergic receptor	Adrenergic receptor	Hs00609046_m1
CHRM2	Muscarinic acetylcholine receptor M2	Acetyhcholine (ACh) receptor	Hs00265208_s1
TNNT2	Cardiac type troponin T2	Cardiac sarcomeric protein	Hs00165960_m1
MYBPC3	Myosin binding protein C, cardiac	Cardiac sarcomeric protein	Hs00165232_m1
CHAT	Choline acetyltransferase	Enzyme catalyzing ACh synthesis	Hs00758143_m1
TH	Tyrosine hydroxylase	Enzyme catalyzing tyrosine to dopamine conversion	Hs00165941_m1
DBH	Dopamine beta-hydroxylase	Enzyme catalyzing dopamine to noradrenaline conversion	Hs01089840_m1
TUBB3	Tubulin beta-3 chain	Microtubular protein	Hs00801390_s1
GAPDH	Glyceraldehyde-3-phosphate dehydrogenase	Housekeeping	Hs02758991_g1
EEF1A1; EE+	Eukaryotic translation elongation factor 1 alpha 1	Housekeeping	Hs00265885_g1
GUSB	Beta-glucuronidase	Housekeeping	Hs00939627_m1
